# Offspring schooling associated with increased parental survival in rural KwaZulu-Natal, South Africa

**DOI:** 10.1016/j.socscimed.2017.01.015

**Published:** 2017-03

**Authors:** Jan-Walter De Neve, Guy Harling

**Affiliations:** a Department of Global Health and Population, Harvard T.H. Chan School of Public Health, 665 Huntington Avenue, Boston, MA 02115, United States; b Institute of Public Health, Heidelberg University, Im Neuenheimer Feld 130.3, Heidelberg 69120, Germany; c Research Department of Infection and Population Health, University College London, off Caper Street, London, WC1E 6JB, United Kingdom; d Africa Health Research Institute, University of KwaZulu-Natal, Somkhele, 3935, South Africa

**Keywords:** Offspring schooling, Survival, South Africa, Longitudinal

## Abstract

**Background:**

Investing in offspring's human capital has been suggested as an effective strategy for parents to improve their living conditions at older ages. A few studies have assessed the role of children's schooling in parental survival in high-income countries, but none have considered lower-resource settings with limited public wealth transfers and high adult mortality.

**Methods:**

We followed 17,789 parents between January 2003 and August 2015 in a large population-based open cohort in rural KwaZulu-Natal, South Africa. We used Cox proportional hazards models to investigate the association between offspring's schooling and time to parental death. We assessed the association separately by parental sex and for four cause of death groups.

**Results:**

A one year increase in offspring's schooling attainment was associated with a 5% decline in the hazard of maternal death (adjusted Hazard Ratio [aHR]: 0.95, 95%CI: 0.94–0.97) and a 6% decline in the hazard of paternal death (aHR: 0.94, 95%CI: 0.92–0.96), adjusting for a wide range of demographic and socio-economic variables of the parent and their children. Among mothers, the association was strongest for communicable, maternal, perinatal and nutritional conditions (aHR: 0.87, 95%CI: 0.82–0.92) and AIDS and tuberculosis (aHR: 0.92, 95%CI: 0.89–0.96), and weakest for injuries. Among fathers, the association was strongest for injuries (aHR: 0.87, 95%CI: 0.79–0.95) and AIDS and tuberculosis (aHR: 0.92, 95%CI: 0.89–0.96), and weakest for non-communicable diseases.

**Conclusion:**

Higher levels of schooling in offspring are associated with increased parental survival in rural South Africa, particularly for mothers at risk of communicable disease mortality and fathers at risk of injury mortality. Offspring's human capital may be an important factor for health disparities, particularly in lower-resource settings.

## Highlights

• Investing in offspring's education may improve parental survival.
• We follow 17,789 parents over a 13 year period in rural South Africa.
• Higher offspring schooling is associated with increased parental survival.
• Strongest association for mothers is for communicable disease mortality.
• Strongest association for fathers is for injury mortality.

## 1 Introduction

Several recent studies from Asia (Cai et al., 2006, Frankenberg et al., 2002, Giles et al., 2011, Knodel et al., 2000), Latin America (Bravo, 2006, Saad, 2005), and sub-Saharan Africa (Adamchak et al., 1991, Kohler et al., 2012) suggest that a majority of individuals of ages 60 and older in low- and middle-income countries depend on the financial support provided by their children. Given the strong empirical relationship between schooling and socioeconomic status, investing in their children's human capital may thus be one of the most effective strategies for parents to improve their living conditions at older ages. This may be particularly the case in settings where public wealth transfers are limited and children play a more prominent role in supporting older generations.

The effect of children's human capital on the health of their parents is likely to be mostly protective for several reasons. First, children may function as a substitute for market institutions (e.g., employer-provided pensions and health insurance) or tax supported institutions (e.g., social security and health insurance) (Barouni and Broecke, 2014, Psacharopoulos, 1994, Schultz, 2004). For example, children could recompense their parents for human capital investments received in childhood (‘parental repayment’), based on their increased financial means arising from their schooling (Clay and Vander Haar, 1993, Frankenberg et al., 2002). Additionally, more highly educated children may be better able to communicate health knowledge and skills acquired at or after school to parents, positively affecting their health behavior (Berkman et al., 2000, Cutler and Lleras-Muney, 2010, Field and de la Roca, 2005, Rowa-Dewar et al., 2014). More educated children may facilitate the use of medical information among their parents (e.g., understanding doctors' prescriptions) or may help their parents navigate the complex web of health insurance bureaucracies (Grossman, 1972). Offspring with additional schooling may also be generally more familiar with modern society (Glewwe, 1999), which may make them more receptive to modern medicine (Aslam and Kingdon, 2012, Frost et al., 2005), or the ‘hidden curriculum’ values of discipline and obedience of authority learned in school (Basu and Stephenson, 2005). Finally, formal schooling may open access to careers in the health sector (e.g., as a nurse practitioner or community health worker), allowing offspring to directly provide care to their parents (Bauman et al., 2006, Evans and Becker, 2009, McGarry, 1998).

While all of these pathways suggest that differential investment in children's human capital may be a key driver of social and health inequalities at older ages (Berkman and Kawachi, 2000, Kaufman and Cooper, 1999, Krieger, 2001, Marmot, 2003), surprisingly little evidence is available on the empirical relationship between children's human capital investment and parental survival. In the United States, one longitudinal study estimated a difference in life expectancy between parents of children with a college degree and parents of children with less than a high school diploma of about two years (age of death 71 vs 69) (Friedman and Mare, 2014). Interestingly, this relationship persisted after controlling for parents' own socio-economic resources, and was more pronounced for deaths that were linked to behavioral factors (most notably, chronic lower respiratory disease and lung cancer). In Sweden, a study compared parental siblings and found that parents whose children had tertiary schooling had a 21% lower hazard of dying compared to their siblings whose children completed only nine years of compulsory schooling (Torssander, 2013). A quasi-experimental study in Sweden, exploiting changes in schooling law as a ‘natural experiment’, found *no evidence* of a causal effect of schooling in children on parental longevity (Lundborg and Majlesi, 2015). Large effects of children's schooling on parental survival might, however, seem unlikely in Sweden – where a comprehensive welfare system generously provides for the elderly, and upward intergenerational wealth transfers are uncommon (Fritzell and Lennartsson, 2005, Lennartsson et al., 2009, Torssander, 2013). Even though children may still provide informal care in this setting (Bolin et al., 2008, Lennartsson et al., 2009), the marginal impact of this additional support in the Swedish setting is likely to be much smaller than the impact in a low-resource setting.

Two relevant panel studies have been conducted in middle-income countries. In a Chinese study, the adjusted hazard of parental death for those living with offspring with 10 or more years of schooling was 17% lower than that for offspring with 6 years of schooling or less (Yang et al., 2016). The study suggests that children's and spousal schooling were similarly important for the health of the elderly in this setting. Another longitudinal study, using data from Mexico, assessed the impact of children's schooling on their parents' functional limitations. In adjusted analysis, parents whose children had completed high school were less likely to report functional limitations than those whose children had not (Yahirun et al., 2016). To our knowledge, no studies have been conducted in sub-Saharan Africa.

### 1.1 South Africa education system

The public education system in South Africa is divided into three phases of basic education (Republic of South Africa, 2013). The ‘foundation’ phase runs from grade R (pre-primary) to grade 3; the ‘intermediate’ phase from grades 4 to 9; and the ‘senior’ phase from grades 10 to 12. Primary school would typically be considered grades 1 to 7, with the *de jure* primary school-age population being 7–13 years old. Lower secondary school would typically be considered grades 8 to 9; upper-secondary school would be considered grades 10 to 12. Students between the ages of 14 and 18 years old are officially being regarded as of secondary school-going age. Schooling is mandatory for children aged 7–15 years (i.e., those in the foundation and intermediate phases). Gross primary school enrollment (both sexes) was 99.7% in 2014 (UNESCO Institute for Statistics, 2016). Gross lower-secondary school enrollment (both sexes) was 94.9% and gross upper-secondary school enrolment (both sexes) was 94.5%. The National Senior Certificate, commonly known as ‘matric’, is a three year qualification that signifies the end of twelve years of schooling. In principle, public schooling is free for those who cannot afford to pay school fees. Tertiary schooling in South Africa offers a wide range of options, including three-year bachelor's degrees and an honors degree, an optional fourth-year qualification, in addition to advanced masters and doctorate degrees.

The relationship between schooling attainment and health outcomes has garnered increasing attention from government, donors, and health system researchers and planners in South Africa. In particular, recent research and policy efforts have increased their focus on improving access and retention in schools as a potential HIV prevention strategy among young women. Two recent RCTs, including in rural KwaZulu Natal (KZN) and Mpumalanga provinces, have assessed the role of schooling conditional cash transfers to reduce HIV infection risk among secondary school students (Karim et al., 2015, Pettifor et al., 2016). Moreover, increased access to, and retention in, school is a key part of the United States President’s Emergency Plan for AIDS Relief DREAMS initiative, which is being piloted in several South African sites (PEPFAR, 2016). The current study thus has important implications for research and policy, in determining whether these efforts to increase school attainment in South Africa may have additional health benefits in terms of increased survival for older generations, over and above those arising more directly from offspring's reduced HIV risk.

We therefore used longitudinal data to test the hypothesis that offspring's schooling decreases the hazard of parental death in an African setting with very high adult mortality (Coovadia et al., 2009). We used one of Africa's largest cohorts, located in rural KZN, South Africa, to follow up 17,789 parents and observe their survival over the period January 2003 to August 2015. The availability of a range of demographic and socio-economic variables in our dataset allowed us to control for important determinants of adult mortality and to assess the role of offspring's schooling on the most prevalent causes of death among their parents in South Africa.

## 2 Methods

### 2.1 Study area

Since 2000, the Africa Centre for Population Health has collected longitudinal demographic, social, and economic data on over 100,000 people living in a 432 km^2^ demographic surveillance area (DSA) in uMkhanyakude District, in northern KZN. The DSA includes both rural areas that were a designated Zulu ‘homeland’ area during Apartheid and urban areas that formerly constituted a black-only township. uMkhanyakude is the poorest of the 11 districts in KZN and the second most deprived district in South Africa (Government of South Africa, 2015). Adult HIV prevalence in this community is very high. Prior to the roll-out of public-sector antiretroviral therapy (ART), adult life expectancy in the DSA was 49.2 (Bor et al., 2013) with over half of the population deaths attributed to HIV (Herbst et al., 2011). Since the roll-out of ART in 2004, however, the burden of HIV on adult mortality in this population is rapidly falling (Reniers et al., 2016). By 2011, adult life expectancy had increased to 60.5 years. All-cause mortality declined by over 50% for adults aged 25 to 44 between 2003 and 2011; however, mortality reductions at older ages were much smaller (Bor et al., 2013).

### 2.2 Data collection

Household surveillance in the DSA began on 1 January 2000. Data on basic household demographics, residence and vital status of DSA household members are collected at least twice a year (Tanser et al., 2008). Individuals are registered if they are a member of a DSA-resident household, even if they are not themselves living in the DSA. Respondents are considered members of the same household regardless of where they live (Hosegood and Timaeus, 2006). This household dataset allowed us to identify any DSA-member child of each registered parent. We had no data, however, on whether parents co-reside with their offspring, or the frequency of their contact. We linked these data from each household visit to individual-level information gathered at annual Household and Socio-Economic Survey (HSE) interviews. The HSE survey (including household wealth quintile) has been conducted annually since 2003, with the exception of 2004 and 2008. Finally, we added detailed information on time and cause of death for deceased individuals, based on verbal autopsy interviews for all notified deaths of DSA household members, which were collected for all survey years (Herbst et al., 2011). Data on our main exposure, highest offspring educational attainment, was missing for 8% of observations for mothers and 7% of observations for fathers. Among parents with complete data on offspring educational attainment, data on our main covariates – marital status, employment status, and household wealth – were available for over 95% of observations, while parental age and area of residence were available for all observations. Data on deaths are based on records for each DSA-registered individual for whom a Death Outcome Notification Form has been recorded. This death notification form triggered the conduct of a verbal autopsy with the closest caregiver of the deceased, on average six months after the death. Verbal autopsy refusals in the DSA were low (2%) (Herbst et al., 2011). Our analysis ran from 1 January 2003 to 16 August 2015.

### 2.3 Sample

Inclusion criteria for our analysis were that a parent had at least one DSA-linked child who was alive at the time of the first household interview and that at least one of these offspring was at least 18 years old. This age restriction was imposed so that offspring had the opportunity to complete secondary schooling or more, which runs until age 18 in South Africa, and so that they were eligible for the survey component on labor market outcomes. Many individuals reside outside the DSA, but continue to be members of a household in the DSA. Exposure continued to accumulate if the individual out-migrated from the DSA but retained membership of a resident household. The ability to track the demographic characteristics of non-resident household members is a major strength of the data. Parents were right-censored if they were lost to follow up due to non-response, lost membership of a resident household, or at the end of the study period (16 August 2015). Participation rates for household data collection were >99%. Previous studies in the DSA have found that attrition due to permanent out-migration or loss to follow-up was low, at a rate of 3.3 per 100 person-years (Bor et al., 2013). Additional details on the cohort are documented elsewhere (Tanser et al., 2008).

### 2.4 Exposure

In our study, we focused on the parent as unit of analysis with the schooling of their offspring as exposure. Our exposure was the highest schooling attainment of any offspring who was a member of a DSA household between 2003 and 2015. School attainment was defined as time-varying total years of schooling completed by the time of each visit. In additional models, we created time-varying indicators for having completed 0–7 years, 8–9 years, 10–12 years, or 13+ years of schooling. We compared parents whose offspring have completed 0–7 years of schooling (i.e., primary schooling or less) to parents whose offspring have completed 8–9 years, 10–12 years, or 13+ years of schooling. These comparisons are likely to be most policy-relevant in South Africa, where much of the current debate is around the benefits of increased access to secondary school and beyond.

### 2.5 Outcome

Our primary outcome was death of the parent. The ‘incidence rate’ of parental death was calculated as the number of parental deaths divided by the total number of person-years of observation between 2003 and 2015, separately for each offspring schooling attainment group. Our secondary outcome of interest was parental cause of death. Causes of death were mapped into Global Burden of Disease groups I (communicable, maternal, perinatal and nutritional conditions), II (non-communicable diseases) and III (injuries)—with the exception of deaths due to tuberculosis (TB) and AIDS. TB and AIDS were classified together into a separate group, given the considerable overlap in mortality from HIV infection and TB (Herbst et al., 2011). Among women in the DSA, the three most common underlying causes of death were lower respiratory infections, meningitis, and diarrheal diseases (group I); cerebro- and cardiovascular diseases, and diabetes mellitus (group II); and violence, road traffic accidents, and other unintentional injuries (group III); in addition to AIDS or TB. The most common underlying causes of death were nearly identical for women and men. Violence, however, caused over 50% of injury-related deaths in men compared to 36% of injury-related deaths in women.

### 2.6 Covariates

**Fig. 1:**
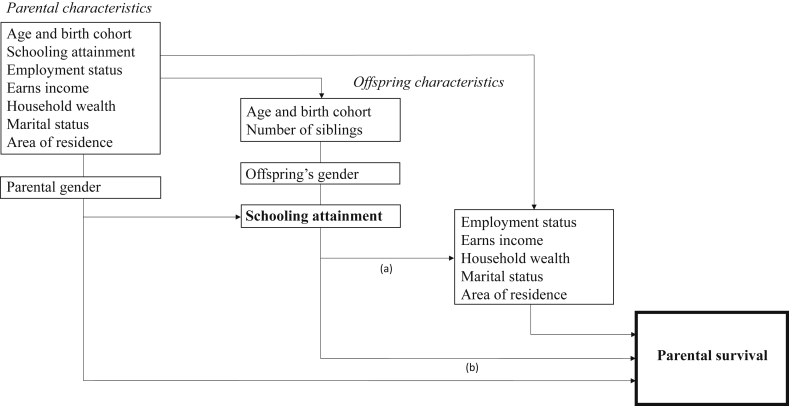
Conceptual hierarchical framework for the relationship between offspring schooling and parental survival. Figure shows the inter-relationship between variables in our study. The arrows represent the potential (causal) effect of the relevant explanatory factor. Offspring schooling attainment exerts its effect on parental survival through: (a) improved labor market outcomes, wealth, assortative mating with better educated spouses, changes in area of residence, and (b) more proximate determinants such as direct transfers of health knowledge and care for parents (Grossman 1972; Mincer, 1974, Bongaarts, 1978, Epstein and Guttman, 1984). In our study, we control for parental and offspring characteristics that are likely confounders, and baseline values of characteristics that are likely on the causal pathway between offspring schooling and parental survival. Our estimates can thus be interpreted as an estimate of the total effects of offspring schooling on parental survival because we allow likely mechanisms to vary during follow-up (pathways a and b).

To account for possible confounding, we included time-varying linear, quadratic and cubic terms in parental age, and a linear term in parental years of schooling, based on tests of functional form (Supplementary Table 1) (Sartorius et al., 2013). We also included time-varying parental marital status (as a binary indicator), number of children (as a continuous variable), age of the oldest child (as a continuous variable), and gender of the oldest child (as a binary indicator) (Downey, 1995, Sun et al., 2015, Tamakoshi et al., 2010). Additionally, we included the baseline values of characteristics that were likely on the causal pathway between offspring schooling and parental survival, to capture the total effects of offspring schooling during follow-up (VanderWeele, 2015). We included the baseline value of employment status of both the oldest child and parent (as a binary indicator), baseline marital status of the oldest child (as a binary indicator), as well as baseline household wealth (quintiles) (Morris, 2000) and baseline household area of residence (urban, periurban, rural) of the parent and their children. We also included indicator variables for year of observation to control for unmeasured confounders that change over time (e.g., changing availability of HIV prevention and treatment services). By simultaneously controlling for year of observation and age, we implicitly also controlled for birth cohort effects. Fig. 1 displays a simplified conceptual framework which underlies our empirical strategy.

### 2.7 Statistical analysis

To investigate the association between parental death and offspring's schooling, we used a Cox proportional hazards model (CPHM). Parents entered the study on 1 January 2003, on their oldest offspring's 18th birthday or at the date of in-migration to the DSA. Parents were followed up until death or right-censoring. In our main analyses, we assessed the incidence of maternal and paternal deaths separately, since mortality risk differs strongly by gender in our study setting, but pooled children of both sexes. In supplementary analyses, we assessed the incidence of parental deaths in subsamples by gender of the oldest child. We verified the proportional-hazards assumption using Schoenfeld residuals (Grambsch and Therneau, 1994). Additionally, we estimated cause-specific models for four common groupings of the most prevalent causes of death in South Africa. The competing causes of deaths are treated as censored observations. Hazard ratios calculated using this approach can be interpreted as ‘among those who did not (yet) experience the competing causes of death’ (Andersen et al., 2012, Lau et al., 2009). All analyses were conducted in Stata 14 (StataCorp, College Station, Texas, USA).

### 2.8 Sensitivity analyses

We conducted a wide range of sensitivity analyses. First, we assessed time to parental death using exponential and Weibull parametric, accelerated failure time (AFT) models. Although parametric models may lead to inconsistent estimates if the baseline function is mis-specified, they will be more efficient than Cox models if the baseline hazard is correctly specified. Second, to allow the baseline hazard to vary by parental age we stratified our analyses by age, since the effect of schooling might differ depending on parental age and when offspring obtained their schooling. Stratified models used the full sample. Third, because grade repetition is high in rural South Africa (Branson et al., 2013) and students may enter formal schooling late, we also assessed the robustness of our results to alternative sample specifications, including the sample of offspring ages either at least 21 or 25 years old. Fourth, we conducted two-level random-intercept Weibull survival models, allowing the effect to vary either: (i) by household membership type of the DSA; or (ii) area of residence of parents (urban, periurban, rural). Finally, we reran our main analyses after multiple imputation of missing variables in the dataset.

## 3 Results

Baseline respondent characteristics are shown in Table 1. Median age at baseline was 49 for mothers and 52 for fathers. Median age at death was 63 (interquartile range [IQR]: 49–76) for mothers and 63 (IQR: 52–74) for fathers. The median years of schooling completed was similar for women and men in our sample (4 [IQR: 0–9] for mothers and 5 [IQR: 0–11] for fathers). Median highest years of schooling among offspring was substantially larger than the schooling attainment of their parents (12 [IQR: 10–12] for offspring of both mothers and fathers). The number of parents whose offspring did not complete any schooling was small (only 285 [2%] mothers and 50 [1%] fathers).

**Fig. 2:**
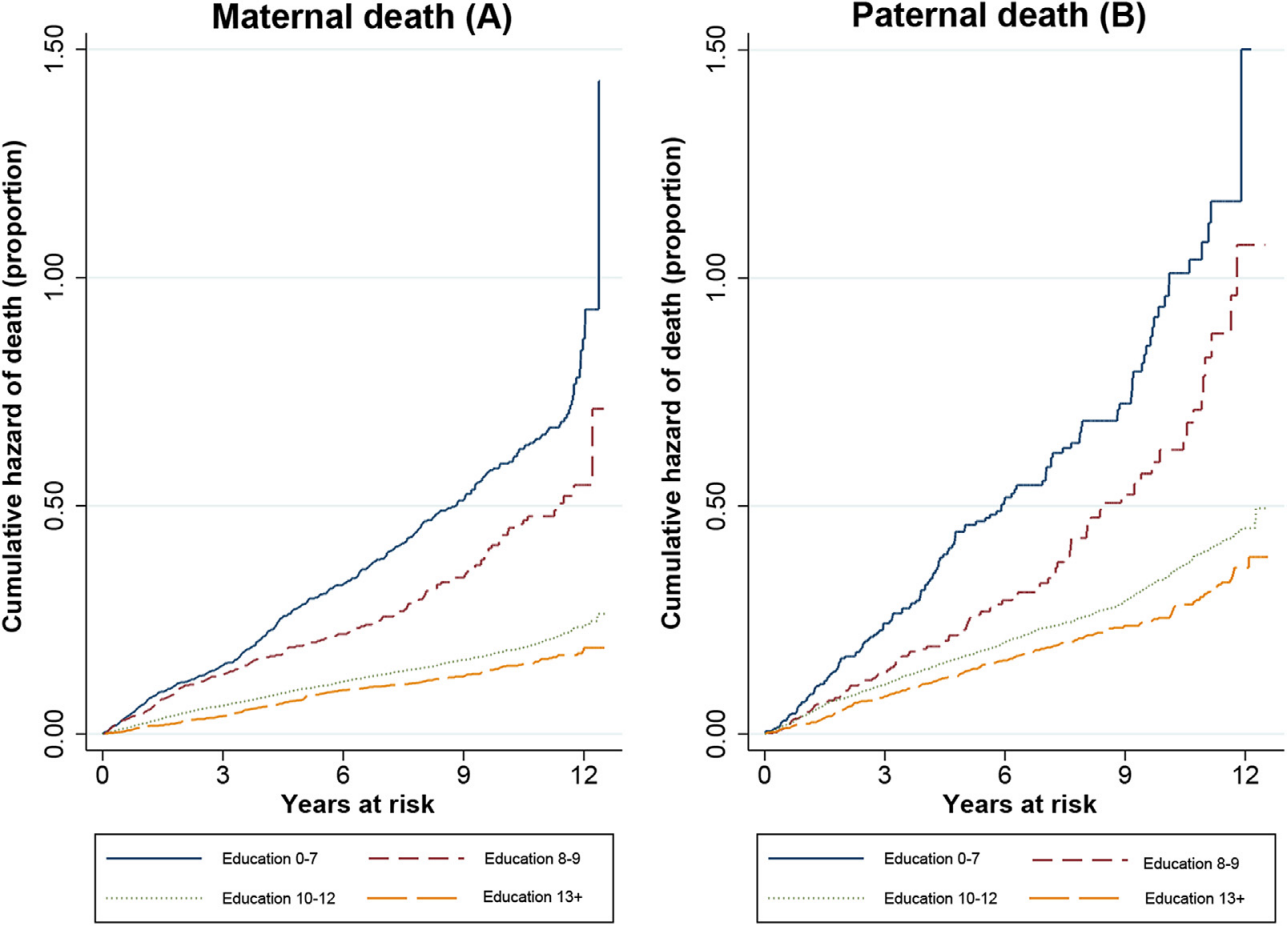
Nelson-Aalen cumulative hazard of parental death, stratified by offspring's schooling.

Between 2003 and 2015, we observed 1886 maternal deaths over 81,653 person-years at risk; and 1344 paternal deaths over 36,001 person-years at risk. The crude incidence of maternal death over the observation period was 2.31 deaths per 100 person-years (95% confidence interval [CI]: 2.21–2.42); whereas the crude incidence of paternal death over the observation period was 3.37 deaths per 100 person-years (95%CI: 3.53–3.93) (Table 2). Incidence was highest for parents of offspring with no schooling: 6.73 maternal deaths per 100 person-years (95%CI: 5.66–8.00) and 12.0 paternal deaths per 100 person-years (95%CI: 8.74–16.51). Fig. 2 shows the cumulative hazard of parental death during follow-up, stratified by offspring's schooling attainment. The cumulative hazard of parental death does not show large deviations from linearity.

In both bivariate and adjusted analysis, parents who had offspring with more years of schooling had significantly lower hazards of death (Table 3). When including offspring's schooling attainment in the model as a continuous variable (model 1), a one year increase in offspring's schooling attainment was associated with a 5% lower hazard of maternal death (adjusted Hazard Ratio [aHR]: 0.95, 95%CI: 0.94–0.97) and a 6% lower hazard of paternal death (aHR: 0.94, 95%CI: 0.92–0.96). When including offspring's schooling attainment levels in the model as indicator variables (model 2), completion of 10–12 years of schooling (versus 0–7 years of schooling) among offspring was associated with a 26% reduction in the hazard of maternal death (aHR: 0.74, 95%CI: 0.64–0.85), and a 35% reduction in the hazard of paternal death (aHR: 0.65, 95%CI: 0.54–0.80). In contrast, lower levels of secondary schooling (8–9 years) were not associated with increased parental survival in adjusted models, suggesting a non-linear relationship in this setting. The relationship was strongest for fathers whose oldest child was male, although child's gender did not have a significant interactive effect (Supplementary Table 3). Supplementary analyses using additional controls and AFT models (Supplementary Table 4), stratified by age (Supplementary Table 5), using alternative sample specifications (Supplementary Table 6), and using multilevel mixed-effects parametric survival models (Supplementary Table 7) yielded similar results. After multiple imputation of missing values, our results remained essentially unchanged (Supplementary Tables 8 and 9).

Table 4 displays results from cause-specific models for common groupings of the most prevalent causes of death. For both mothers and fathers in our analytical sample, NCDs were the most common cause of death, followed by AIDS or TB. Parents of offspring with more schooling were less likely to die of communicable diseases (mothers) and injuries (fathers) in particular. A one-year increase in offspring's schooling attainment was associated with a 13% decline (aHR: 0.87, 95%CI: 0.82–0.92) in maternal hazard of death due to CPMN and a 13% decline (aHR: 0.87, 95%CI: 0.79–0.95) in paternal hazard of death due to injury. Similarly, one additional year of offspring schooling was associated with a 8% decline (aHR: 0.92, 95%CI: 0.89–0.96) in maternal and paternal hazard of death due to AIDS or TB. Parents of offspring with more schooling were also less likely (to a smaller extent) to die as a result of NCDs. Mothers of offspring with more schooling were not less likely to die as a result of injury, although the number of injury deaths among mothers was low (50 maternal cases vs. 92 paternal cases).

## 4 Discussion

Using a large prospective South African cohort, we found that additional schooling in offspring is associated with significantly increased parental survival. Overall, the association was similarly strong for mothers as for fathers. This relationship persisted after controlling for a range of known determinants of adult mortality in rural South Africa, such as age, schooling, marital status, area of residence, employment status, and household wealth of both the parent and offspring. We also controlled for unmeasured confounders that change over time, such as the roll-out of HIV prevention and treatment in South Africa. Our results were robust across a wide range of additional sensitivity analyses, including alternative functional forms of parental age and schooling, and alternative specifications of the baseline hazard function. A handful of previous studies have assessed the role of offspring's schooling in parental survival, but these studies have generally used data from developed countries (Friedman and Mare, 2014, Lundborg and Majlesi, 2015, Torssander, 2013, Zimmer et al., 2007). This study is among the first to extend this growing literature to a lower-resource setting with limited social welfare systems and very high adult mortality.

A second key finding of our study is that higher levels of schooling (i.e., upper secondary school and beyond) among offspring appeared to drive the survival benefit for parents. Late secondary and tertiary education may be particularly important for several reasons: the development of new skills (e.g., complex reasoning and future-oriented thinking) with strong returns in the labor market (Crews et al., 2007, Dahl, 2004, Steinberg, 2005) and the decisions facing adolescents have significant path dependence, such as childbearing and planning a career path (Wilde et al., 2010). These results suggest that the years of late secondary and tertiary education may be a ‘critical period’ (Cunha and Heckman, 2007) to invest in the human capital of children for parental health.

While no previous studies have examined sub-Saharan Africa, our results are consistent with recent studies that found a strong protective association between offspring's schooling and: (i) parental mortality in China (Yang et al., 2016); and (ii) parental functional limitations in Mexico (Yahirun et al., 2016). Interestingly, all three settings share some characteristics with higher-resource countries (e.g., a relatively large elderly population) and some with lower-resource settings (e.g., limited social welfare). The role of offspring's schooling in low- and middle-income countries is likely to be of particular importance, since a larger proportion of the elderly depend on their children for financial support and co-residence of parents with offspring is more common (Cai et al., 2006, Giles et al., 2011). Faced with an increasingly large elderly population, human capital investments in younger populations in lower-resource settings may pay important dividends to older generations.

Our findings are particularly relevant to other sub-Saharan African countries, where supply-side education reforms have greatly increased access to schooling in recent decades. Zimbabwe, for instance, rapidly expanded access to secondary schools for black citizens in the 1980s through a government-initiated school construction program focused on rural areas (Ansell, 2002). Each additional year of maternal schooling induced by the reform reduced mortality among their offspring by about 21% (Grépin and Bharadwaj, 2015). Our findings suggest that additional schooling in children may not only generate such large ‘downstream’ benefits for the next generation, but could also have ‘upstream’ impacts on the preceding one. Further investigation of the impact of expanding schooling access on the health of family members in sub-Saharan Africa appears warranted.

We also assessed the association between offspring's schooling and parental causes of death, grouped by GBD categories. Additional schooling in offspring was associated with the largest reduction in parental death for communicable diseases (mothers) and injury (fathers). The strong association between offspring schooling and reduced risk of death due to injury among their fathers (but not mothers) is of particular interest. One possible explanation for this finding might be that children share information acquired at school with their fathers on the health risks associated with driving under influence of alcohol or share norms and values with their fathers around violence, possibly leading to fewer injuries. While these examples seem entirely plausible, one would expect deaths due to injury to more often occur as a result of external circumstances as opposed to individual behavioral factors such as those linked to AIDS, TB, or NCDs. Whether these results for injury are due to residual confounding, however, cannot be ascertained with the current study design.

Among both mothers and fathers, a strong association was also seen for death due to AIDS or TB. The difference seen between results for AIDS or TB compared to NCDs suggests a potentially important role for additional human capital in children in a setting where HIV is endemic. Exposure to additional school-based HIV prevention programming may have changed knowledge and attitudes (e.g., on the benefits of HIV testing, early ART initiation or adherence) (Gallant and Maticka-Tyndale, 2004). Children may also have benefited from HIV education in the broader school curriculum, insofar as this latter had changed since their parents were at school. Since the government of South Africa provides an old-age pension to the elderly (Case and Deaton, 1998), non-pecuniary pathways (such as HIV knowledge and attitudes) may play a more important role relative to wealth transfers than might be seen in other sub-Saharan settings. Conversely, this smaller role for wealth transfers in South Africa suggests that the protective effect of offspring education on parental health outcomes may be even stronger elsewhere in sub-Saharan Africa.

Finally, we note that parental risk-profiles and survival depend on the setting under study. Previous studies from more developed settings have examined samples of parents that were much older: at least 50 years old in the United States and Western-European countries (Friedman and Mare, 2014, Sabater and Graham, 2016), at least 60 years old in Sweden and Taiwan (Lundborg and Majlesi, 2015, Torssander, 2014, Zimmer et al., 2007) and at least 65 years old in China (Yang et al., 2016). In contrast, the results presented here reflect the survival of parents that are on average much younger (median of 49 for women and 52 for men) and die much earlier (median of 63 for both women and men). Nevertheless, given the lower life expectancy in South Africa relative to other countries studied, this population was not unusually early in their life course. In this study's surveillance area, between 2000 and 2009, HIV-related causes constituted the largest cause-specific mortality fraction among those ages 50–64 (42%, as opposed to 27% due to lifestyle-related NCDs) (Herbst et al., 2011). These parents were at particular risk of mortality due to HIV-related causes, but relatively young to die of NCDs. An additional implication is that reduced mortality among these younger middle-aged parents is likely to have large additional positive effects on labor market outcomes (e.g., younger and healthier parents could return to work (Bor et al., 2012)), and other social outcomes.

### 4.1 Limitations

A great strength of our analysis was the availability of time and cause of death of parents, which allowed us to assess the differential role of offspring's schooling for the most prevalent causes of death in a population-based cohort in South Africa. Nevertheless, our study has some limitations. First, although we control for a wide range of covariates available in the dataset, the design of our study unfortunately does not allow for causal inference. There may be confounding by factors which we were unable to control for (e.g., unmeasured parental attitudes or resources), in which case offspring's schooling may be a strong marker of parental survival, as opposed to an exposure. Given the scarcity of evidence on the role of human capital in children and parental old-age survival, however, the aim of this study was to extend the current associational literature to low-resource settings with high mortality, to serve as a comprehensive baseline analysis for subsequent (quasi-)experimental research. Future research, for instance, could exploit schooling policy reforms in sub-Saharan Africa as a ‘natural experiment’ (De Neve et al., 2015, Grépin and Bharadwaj, 2015). Future research could also use mediation analysis methods to examine the proportions of the association between offspring schooling and parental survival that were mediated by employment status and household wealth (VanderWeele, 2015), conduct additional sensitivity analyses to assess the robustness of our findings (Ding and VanderWeele, 2016), or conduct intervention studies to test (specific pathways of) the effect of offspring schooling on parental survival.

Second, as with any long-term community-based study, the cohort suffered from attrition and non-response. However, non-response was very limited (household participation rates were >99%) and our results did not change after accounting for data missingness through multiple imputation. Third, there may be mismeasurement in our exposure and outcome. During verbal autopsies, for instance, caregivers may refuse or seek to underreport deaths due to HIV out of fear of HIV-related stigma in the community. Refusals to report deaths, however, were low (2%), and verbal autopsies were conducted by nurses with extensive training in administering the questionnaire (Herbst et al., 2011). Fourth, since our study is focused on one geographically limited population, our results may not generalize widely. Educational attainment among offspring in our study, for instance, was relatively high (median years of schooling was 12) and similar for girls and boys. Our findings may not generalize to settings where educational opportunities are more limited, or more gender-unequal. Our findings are likely informative, however, of the association between offspring's schooling and parental old-age survival in low-resource settings, particularly where HIV is endemic.

## 5 Conclusions

Additional schooling in offspring is associated with increased parental survival in rural South Africa, particularly for mothers at risk of communicable disease mortality and fathers at risk of injury mortality. These findings suggest that schooling may not only have large benefits for those enrolled, but could also be beneficial for older family members. Studies focusing only on the private returns to schooling may underestimate the societal benefits, particularly in settings where welfare sectors are underdeveloped and adult mortality is high.

## Figures and Tables

**Table 1:** Baseline characteristics of the study sample (complete case dataset).

	Mothers	Fathers
No. of mothers/fathers	12,105	5684
No. of subsequent maternal/paternal deaths	1886	1344
Age (years)	49 (43–61)	52 (47–62)
Schooling (years)	4 (0–9)	5 (0–11)
Highest schooling attainment		
0 years	3981 (33)	1803 (32)
1–7 years	4189 (35)	1749 (31)
8–9 years	924 (8)	417 (7)
10–12 years	2006 (17)	923 (16)
13+ years	1005 (8)	792 (14)
Household wealth quintile		
Lowest	3076 (25)	1319 (23)
2nd lowest	3101 (26)	1475 (26)
Middle	2335 (19)	1074 (19)
2nd highest	1800 (15)	828 (15)
Highest	1793 (14)	988 (17)
Married	3773 (31)	3843 (68)
Area		
Rural	7802 (64)	3972 (70)
Peri-urban	3523 (29)	1417 (25)
Urban	780 (6)	295 (5)
No. of children	3 (2–5)	4 (2–5)
Offspring's highest schooling (years)	12 (10–12)	12 (10–12)
Offspring's highest schooling attainment		
0 years	285 (2)	50 (1)
1–7 years	937 (8)	291 (5)
8–9 years	1061 (9)	508 (9)
10–12 years	7883 (65)	3922 (69)
13+ years	1939 (16)	913 (16)

Figures for categorical data are N (%); figures for continuous data are medians and interquartile ranges in parentheses.

**Table 2:** Sample characteristics of parents followed over time.

	Person-years	% of person-years	Parental deaths (cases)	Incidence rate (per 100 person-years)
*Maternal mortality*				
All	81,653	100.0	1886	2.31 (2.21–2.42)
Offspring's highest schooling attainment				
0 years	1902	2.3	127	6.73 (5.66–8.00)
1–7 years	5063	6.2	287	5.67 (5.05–6.36)
8–9 years	4670	5.7	199	4.26 (3.71–4.90)
10–12 years	53,043	65.0	1022	1.93 (1.81–2.05)
13+ years	16,975	20.8	251	1.48 (1.31–1.67)

*Paternal mortality*				
All	36,001	100.0	1344	3.37 (3.53–3.93)
Offspring's highest schooling attainment				
0 years	316	0.9	39	12.0 (8.74–16.51)
1–7 years	1311	3.6	108	8.23 (6.82–9.94)
8–9 years	1946	5.4	107	5.50 (4.55–6.64)
10–12 years	24,442	67.9	865	3.54 (3.31–3.78)
13+ years	7985	22.2	225	2.82 (2.47–3.21)

The sample included 12,105 mothers and 5684 fathers followed over the years 2003–2015. Results for additional variables are shown in Supplementary Tables.

**Table 3:** Survival models showing the association between offspring's schooling and hazard of parental death.

	Bivariate analysis HR (95% CI)	Multivariable analysis aHR (95% CI)
*Maternal hazard of death*		
Model 1: Offspring highest schooling (years)	0.88 (0.87–0.89)	0.95 (0.94–0.97)
Model 2: Offspring's highest schooling attainment		
0–7 years	1	1
8–9 years	0.71 (0.60–0.84)	1.08 (0.90–1.29)
10–12 years	0.32 (0.29–0.36)	0.74 (0.64–0.85)
13+ years	0.25 (0.21–0.29)	0.58 (0.48–0.70)

No. of mothers	12,105	12,105

*Paternal hazard of death*		
Model 1: Offspring highest schooling (years)	0.88 (0.87–0.90)	0.94 (0.92–0.96)
Model 2: Offspring's highest schooling attainment		
0–7 years	1	1
8–9 years	0.60 (0.48–0.78)	0.86 (0.66–1.11)
10–12 years	0.39 (0.32–0.46)	0.65 (0.54–0.80)
13+ years	0.30 (0.25–0.38)	0.57 (0.44–0.72)

No. of fathers	5684	5684

Output from separate multivariable survival models including model 1 (continuous variable for schooling attainment) and model 2 (indicator variables for schooling attainment levels). HR: hazard ratio; aHR: hazard ratio adjusted for a linear, quadratic and cubic term in paternal age, parental years of schooling, parental marital status, number of children, age of the oldest child, gender of the oldest child, indicators for year of observation, baseline marital status of the oldest child, baseline employment status of the oldest child and parent, and baseline household wealth and area of residence (rural, peri-urban, urban). CI: confidence interval. Results for additional variables are shown in Supplementary Tables.

**Table 4:** Output from separate multivariable survival models by parental cause of death.

Cause of death	Non-communicable	AIDS or tuberculosis	Communicable, maternal, perinatal or nutritional	Injuries
	aHR (95% CI)	aHR (95% CI)	aHR (95% CI)	aHR (95% CI)
*Maternal hazard of death*				
Model 1: Offspring highest schooling (years)	0.97 (0.95–1.00)	0.92 (0.89–0.96)	0.87 (0.82–0.92)	0.96 (0.87–1.05)
Model 2: Offspring's highest schooling attainment				
0–7 years	1	1	1	1
8–9 years	1.06 (0.81–1.38)	1.20 (0.84–1.70)	0.85 (0.45–1.63)	0.34 (0.07–1.58)
10–12 years	0.85 (0.70–1.03)	0.76 (0.56–1.03)	0.38 (0.22–0.65)	0.63 (0.27–1.49)
13+ years	0.75 (0.58–0.96)	0.42 (0.28–0.65)	0.21 (0.10–0.47)	0.51 (0.17–1.60)

No. of mothers	12,105	12,105	12,105	12,105
Maternal deaths (cases)	908	564	125	50

*Paternal hazard of death*				
Model 1: Offspring highest schooling (years)	0.95 (0.92–0.98)	0.92 (0.89–0.96)	0.93 (0.86–1.01)	0.87 (0.79–0.95)
Model 2: Offspring's highest schooling attainment				
0–7 years	1	1	1	1
8–9 years	0.99 (0.66–1.02)	0.59 (0.36–0.95)	1.10 (0.46–2.63)	1.04 (0.39–2.76)
10–12 years	0.76 (0.56–1.02)	0.62 (0.44–0.89)	0.64 (0.33–1.27)	0.38 (0.16–0.89)
13+ years	0.57 (0.40–0.82)	0.46 (0.29–0.73)	0.52 (0.21–1.31)	0.65 (0.24–1.73)

No. of fathers	5684	5684	5684	5684
Paternal deaths (cases)	569	427	88	92

Output from separate multivariable survival models including model 1 (continuous variable for schooling attainment) and model 2 (indicator variables for schooling attainment). HR: hazard ratio; aHR: hazard ratio adjusted for a linear, quadratic and cubic term in paternal age, parental years of schooling, parental marital status, number of children, age of the oldest child, gender of the oldest child, indicators for year of observation, baseline marital status of the oldest child, baseline employment status of the oldest child and parent, and baseline household wealth and area of residence (rural, peri-urban, urban). CI: confidence interval.
