# Supplementary material for: Offspring schooling associated with increased parental survival in rural KwaZulu-Natal, South Africa
Source: Soc Sci Med. 2017 Mar;176:149–57. doi: 10.1016/j.socscimed.2017.01.015 (PMC5322823; doi:10.1016/j.socscimed.2017.01.015)
Supplement: Supplementary Tables [file mmc1.docx]

# Supplementary materials

In this supporting materials appendix to the manuscript “Offspring schooling associated with increase in parental survival in rural KwaZulu-Natal, South Africa”, we provide Supplementary Tables 1 – 9.

Supplementary Table 1: Cox proportional hazards models for appropriate functional form (hazard ratio, 95%CI)

## Supplementary Table 2a: Sample characteristics of mothers followed over time

## Supplementary Table 2b: Sample characteristics of fathers followed over time

## Supplementary Table 3: Cox proportional hazards models for parental hazard of death in subsamples by gender of the oldest child

## Supplementary Table 4: Sensitivity analyses for Cox proportional hazards models

## Supplementary Table 5: Cox proportional hazards models for parental hazard of death stratified by parental and child age

## Supplementary Table 6: Cox proportional hazards models for parental hazard of death using alternative sample specifications

## Supplementary Table 7: Two-level random-intercept survival models using data on household membership type and area of residence

## Supplementary Table 8: Descriptive statistics for multiple imputation

## Supplementary Table 9a: Regression results from multiply imputed dataset for maternal death (hazard ratios and 95% confidence intervals)

## Supplementary Table 9b: Regression results from multiply imputed dataset for paternal death (hazard ratios and 95% confidence intervals)
